# 3D ZnO/Activated Carbon Alginate Beads for the Removal of Antibiotic-Resistant Bacteria and Antibiotic Resistance Genes

**DOI:** 10.3390/polym15092215

**Published:** 2023-05-07

**Authors:** Zhe Liu, Xi Yu, Zhenchao Zhou, Jinyu Zhou, Xinyi Shuai, Zejun Lin, Hong Chen

**Affiliations:** 1 College of Environmental and Resource Sciences, Zhejiang University, Hangzhou 310058, China; 22014059@zju.edu.cn (Z.L.); 22114054@zju.edu.cn (J.Z.);; 2 International Cooperation Base of Environmental Pollution and Ecological Health, Science and Technology Agency of Zhejiang, Zhejiang University, Hangzhou 310058, China; 3 Key Laboratory of Environment Remediation and Ecological Health, Ministry of Education, College of Environmental Resource Sciences, Zhejiang University, Hangzhou 310058, China

**Keywords:** antibiotic resistance, calcium alginate, zinc oxide/activated carbon, fixed bed, reactive oxygen species

## Abstract

The worldwide prevalence of antibiotic-resistant bacteria (ARB) and antibiotic resistance genes (ARGs) have become one of the most urgent issues for public health. Thus, it is critical to explore more sustainable methods with less toxicity for the long-term removal of both ARB and ARGs. In this study, we fabricated a novel material by encapsulating zinc oxide (ZnO) nanoflowers and activated carbon (AC) in an alginate biopolymer. When the dosage of ZnO was 1.0 g (≈2 g/L), the composite beads exhibited higher removal efficiency and a slight release of Zn^2+^ in water treatment. Fixed bed column experiments demonstrated that ZnO/AC alginate beads had excellent removal capacities. When the flow rate was 1 mL/min, and the initial concentration was 10^7^ CFU/mL, the removal efficiency of ARB was 5.69-log, and the absolute abundance of ARGs was decreased by 2.44–2.74-log. Moreover, the mechanism demonstrated that ZnO significantly caused cell lysis, cytoplasmic leakage, and the increase of reactive oxygen species induced subsequent oxidative stress state. These findings suggested that ZnO/AC alginate beads can be a promising material for removing ARB and ARGs from wastewater with eco-friendly and sustainable properties.

## 1. Introduction

Since their discovery in the 1940s, antibiotics have been widely used in aquaculture, livestock breeding, and controlling human infectious diseases [1,2,3]. However, the abuse and overuse of antibiotics have caused the progressive increase of antibiotic-resistant bacteria (ARB) and antibiotic resistance genes (ARGs), which have led to high biological risks worldwide [4,5,6]. Owing to their potential risks, ARB and ARGs are characterized as emerging environmental contaminants [7] and recognized as one of the three severe threats to public health [8].

Conventional disinfection, such as chlorination and ultraviolet, are mostly used in wastewater treatment [9,10,11]. Nevertheless, previous studies have reported that these methods have limited efficiency in removing ARGs [12,13], forming disinfection byproducts [14], and inducing ARB to enter viable but nonculturable states [15]. Therefore, it is essential to develop efficient and eco-friendly disinfection methods. To date, metal oxides, including TiO_2_ and ZnO, have been frequently reported because of their bactericidal properties without any toxic byproducts [16]. Compared to the other reported materials, ZnO has more advantages, such as simple preparation, low cost, and high stability [17]. Recently, the performance of ZnO was optimized by modifying its structure [18] or exploring green synthesis methods [19]. However, in the case of water treatment, ZnO still exhibits issues such as limitation of aggregation, difficulty in recycling, and high leaching of Zn^2+^ into water [20]. To address these issues, biopolymer matrices of alginate or chitosan, which were extracted from nature with non-toxicity and biodegradability, were introduced to immobilize and slow the leaching of metal ions [21,22,23], while activated carbon (AC) was added to enhance mechanical strength and adsorption capacity [24].

Some researchers have investigated the potential of ZnO-alginate beads for disinfection treatment in water [25,26]. In contrast, a few reports have studied the efficiency of the composite in removing ARB and ARGs. In addition, few reports have been made on fixed beds to remove ARB and ARGs, and fixed bed study is a fundamental step for engineered applications. In this context, column experiments with the composite were designed and used for efficient removal under the influence of bed depths, initial bacterial concentrations, and flow rates. Considering technologies for low-cost and point-of-use treatment [27], these filtration and disinfection systems are expected to remove ARB and ARGs from highly contaminated wastewater independently without extra energy input.

In this paper, synthesized ZnO nanoflowers and AC were encapsulated in sodium alginate to form composite beads. This study aimed to (1) characterize the alginate composites and compare the performance of ZnO/AC alginate beads against a model ARB *E. coli* HB101, (2) examine the efficiency of removing ARB and ARGs of fixed bed with different parameters, and (3) illustrate the possible mechanism of ARB removal.

## 2. Materials and Methods

### 2.1. Materials

ZnO nanoflowers were simply prepared through a one-step hydrothermal method published by Xu [28]. Briefly, 2.5 mmol zinc acetate dihydrate and 2.5 mmol sodium citrate were dissolved in deionized (DI) water with the addition of 4.0 M NaOH. Then, the above mixture was transferred into a 100 mL Teflon-lined autoclave and heated at 120 °C for 8 h. The industrial ZnO (99.99% purity) was purchased from Aladdin (Hangzhou Bangyi Chemical Co., Ltd., Hangzhou, China). Sodium alginate (CP) was purchased from SCR Co., Ltd. (Shanghai, China), and activated carbon (AR, 200 mesh) was purchased from Xianding Biotechnology Co., Ltd. (Shanghai, China). The model antibiotic-resistant bacteria strain was *E. coli* HB101 which carried three plasmid-encoded ARGs (*tet*A, *bla*_TEM_, and *aph*(3′)-Id).

### 2.2. Synthesis of ZnO/AC Alginate Beads

The different amounts of ZnO (0 g, 0.25 g, 0.5 g, and 1 g) and 0.2 g AC (2 g/L), the typical level used in previous studies, were added to 100 mL of DI water. Then, sodium alginate (2.5% *w*/*v*) was mixed with the above solution under ultrasonic radiation and stirred until a homogeneous mixture was obtained. After that, uniform ZnO/AC-alginate solution was injected into 0.3 M CaCl_2_ solution dropwise to form crosslinked hydrogel beads (~3 mm). Subsequently, the yielded beads were left in the solution for 2 h to harden and then washed thoroughly to remove residual surface reagents. Afterward, these ZnO/AC alginate beads were stored in sealed and sterilized containers at room temperature.

### 2.3. Characterization of ZnO/AC Alginate Beads

X-ray diffraction (XRD, Bruker D8 Advance, Karlsruhe, Germany) and Fourier-transform infrared spectroscopy (FTIR, Nicolet iS50FT-IR, Madison, WI, USA) patterns were recorded to investigate the crystal structure and the functional groups of the synthesized material. The morphology and elemental components were observed using scanning electron microscopy (SEM, Hitachi SU8010, Tokyo, Japan) and energy dispersive spectroscopy (EDS). The UV−vis absorption spectra were estimated using a spectrophotometer (DR 5000, Hach, Loveland, CO, USA), and the fluorescence emission spectra of samples was recorded using a Fluorescence spectrometer (FLS1000, Edinburgh, UK).

To examine the release of Zn^2+^ ions, samples were prepared after mixing 10 g beads with 100 mL DI water while containers shaking at 37 °C for 240 min and collected at time intervals. The Zn content was precisely analyzed with ICP-MS (PerkinElmer, NexION 300X, Waltham, MA, USA).

### 2.4. The Removal Efficiency of ARB by ZnO/AC Alginate Beads

A single colony of *E. coli* HB101 was inoculated in LB Broth for 16 h overnight at 37 °C to reach on log phase. Bacteria were then harvested by centrifuging and resuspended in 0.01 M phosphate buffer solution (PBS, pH = 7.2) to maintain OD_600_ around 0.6. At this moment, the concentration of bacteria was measured using the cell counting method at 10^8^ CFU/mL. Subsequently, the bacteria were mixed with different ZnO content (0 g, 0.25 g, 0.5 g, and 1 g) and AC content (0 g, 0.2 g) of beads. Then an aliquot of 100 μL was withdrawn every 2 h. The treated bacterial suspension was serially diluted to 10-fold increments using PBS and coated onto selective LB agar plates containing tetracycline, kanamycin, and ampicillin. Removal difference was identified using the cell counting method, and the above experiments were done in triplicate.

### 2.5. Application of ZnO/AC Alginate Beads in Fixed Bed

Fixed bed experiments were conducted in glass columns, provided with an inlet, outlet, and glass valve, with an internal diameter of 22 mm and length of 300 mm. Before treatment, the glass tube, valve, inlet pipe, and packing tools were sterilized using UV irradiation of clean bench. The bed depth was designed for 10 cm, while the removal efficiency was investigated under different initial concentrations (10^8^, 10^7^, 10^6^ CFU/mL) with a hydraulic retention time (HRT) of 0.32 h and at varying flow rates (1, 2, 3 mL/min) with HRT of 0.63 h, 0.32 h, and 0.21 h, respectively. Each fixed bed unit was operated in downflow mode, and bacteria suspension flow was continuously driven by gravity. The number of colony-forming units (CFU) per mL of effluent was recorded using the cell counting method.

Breakthrough experiments were described as a ratio of N_t_/N_0_ where N_0_ and N_t_ refer to the concentrations of influent and effluent at time t, respectively. The breakthrough point is when bacteria at the outlet reaches 10% (N_t_/N_0_ ≈ 0.1) of the initial concentration. Therefore, the scatter graph of (N_t_/N_0_) versus t was plotted to visualize the performance. *AEC* interprets the treated capacities against contaminated influent before the materials were exhausted, which was calculated using Equation (1):

(1) $$AEC=\frac{M}{V_{t}}$$

where *M* represents the mass of dried material in g and *V_t_* is the influent volume in L.

### 2.6. Absolute Abundance of Antibiotic Resistance Genes

Samples were collected from the outlet of the fixed bed and then filtered through 0.22 μm aquo-filter membranes. DNA has been extracted using FastDNA SPIN Kit (MP Biomedicals, Santa Ana, CA, USA) and measured on NanoDrop LITE (Thermo, Waltham, MA, USA). The removal efficiency of ARGs was detected by quantitative real-time PCR (qPCR) using the Bio-Rad iQ5 (Bio-Rad, Hercules, CA, USA). The information of primers and standard curves of *tet*A, *bla*_TEM_, and *aph*(3′)-Id were shown in Table S1. The qPCR system of 15 μL contains 1 μL of DNA template, 7.5 μL of SYBR Premix Ex Taq (TaKaRa, Dalian, China), 5.5 μL distilled H_2_O and 0.5 μL of each primer. After the reaction program that followed, the absolute abundance of gene copy number was calculated.

### 2.7. Mechanism of ARB Removal

#### 2.7.1. Microbial Morphology Using SEM

Microstructure before and after the exposure for 120 min was observed using SEM to verify the progressive cellular damage of ARB. The precipitation of bacteria was collected and sealed with 2.5% glutaraldehyde at 4 °C overnight. Afterward, the pretreatment and details of sample detection were conducted as a laboratory manual.

#### 2.7.2. Live/Dead Fluorescence Assay

Bacterial viability was tested using confocal laser scanning microscopy (LSM880, Dresden, Germany) using the LIVE/DEAD BacLight Bacterial Viability Kit (L7012, Thermo Fisher, Waltham, MA, USA). As per the operation manual, a 3 μL mixture (1:1) of fluorescent nucleic acid dye (SYTO 9/PI) was reacted with 1 mL bacterial suspension in the dark for 15 min. Then, samples were identified with excitation/emission wavelengths of 488/537 nm and 561/624 nm.

#### 2.7.3. Lipid Peroxidation Analysis

Malondialdehyde (MDA), which is the biomarker of lipid peroxidation reaction, was detected according to the protocol of the MDA test kit (Solarbio, Beijing, China) using thiobarbituric acid reactive substances assay (TBARS). Samples were collected at 0.5 h, 2 h, and 4 h, and the absorbance was recorded at 532 nm and 600 nm.

#### 2.7.4. Oxidative Stress Measurements

The oxidative stress of bacteria was evaluated using intracellular reactive oxygen species (ROS) and the activity of corresponding antioxidant enzymes. Intracellular ROS was analyzed using the fluorescent probe of 2′,7′-dichlorofluorescein diacetate (DCFH-DA) (EMD Millipore, Burlington, MA, USA), which can be oxidized to fluorescent dichlorofluorescein [29]. Flow cytometry (FCM, BD FACSVerse, Piscataway, NJ, USA) was employed to monitor the fluorescence intensity of 20,000 cells under wavelengths of 488 nm and 525 nm. The activities of superoxide dismutase (SOD) and catalase (CAT) were measured using detection kits (Solarbio, Beijing, China) based on absorbance at 560 nm and 240 nm via a multimode reader (VLBL00D1, Thermo Fisher, Waltham, MA, USA).

### 2.8. Statistical Analysis

All the results were analyzed and calculated as mean and deviation values (n ≥ 3) using Microsoft Excel 2016. Data visualization was completed using Origin 2022b (Origin Lab Corporation, Northampton, MA, USA), and significant differences were conducted in SPSS V24 (IBM, Armonk, NY, USA) at two levels (* *p* < 0.05; ** *p* < 0.01).

## 3. Results and Discussion

### 3.1. Characterization of ZnO/AC Alginate Beads

**Figure 1:**
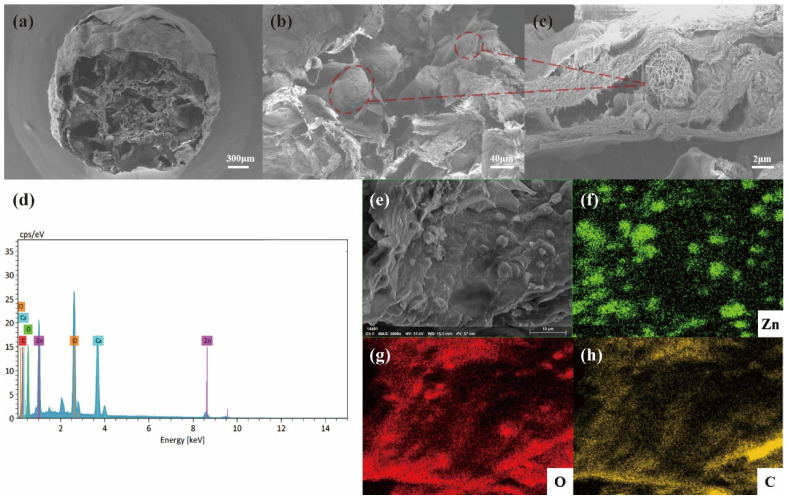
SEM images of (**a**) low magnification and (**b**,**c**) high magnification of the composite. (**d**) EDS analysis, (**e**) HAADF image and elemental mapping of Zn (**f**), O (**g**), and C (**h**).

SEM and EDS analysis were used to detect the morphology, size of the composite, and dispersion of particles. Based on the SEM images, the diameter of ZnO/AC coated in alginate beads was around 2 mm with a porous mesh structure after freeze-drying (Figure 1a). ZnO nanoflowers exhibited uniform growth on the surface and between the porosity (Figure 1b), as shown in high magnification (Figure 1c). As shown in Figure S1, the diameter of synthesized ZnO was about 3.8 μm, as assembled from nanosheets of 30–40 nm in thickness. As shown in Figure S1c, the bactericidal activity of synthesized ZnO was also superior to that of industrial ZnO. From EDS analysis (Figure 1d–h), the elements of Zn, C, and O were represented as clear peaks, and other elements like Cl and Ca were the main elements of the solution during crosslinking. The Zn, C, and O elements were also observed in elemental mapping images, further demonstrating the uniform distribution of ZnO and AC in the composite.

**Figure 2:**
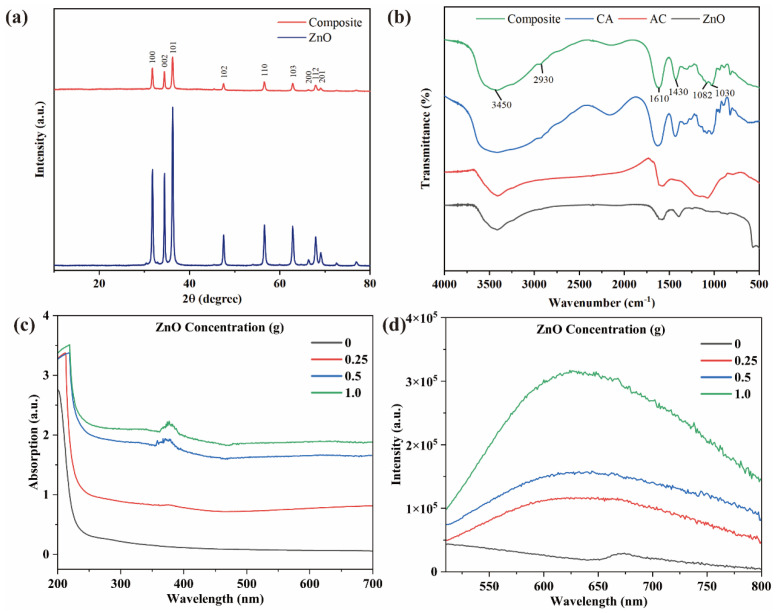
(**a**) XRD patterns and (**b**) FTIR spectra. (Composite = the composite beads; CA = calcium alginate; AC = activated carbon; ZnO = synthesized ZnO). (**c**) UV−vis absorption spectra (**d**) The fluorescence (FL) intensity.

XRD patterns of self-assembled ZnO and the composite beads are shown in Figure 2a. The weakened peaks of bead powder can be attributed to the introduction of calcium alginate and AC, while other diffraction peaks corresponded to ZnO (JCPDS 36–1451). The FTIR spectra of the composite, calcium alginate, AC, and ZnO were recorded in Figure 2b which composite beads exhibited O–H stretching vibration and –COO– stretching vibration absorption peaks. In addition, C–O–C and –C–O–Ca–O–C–O– at 1030 cm^−1^ were attributed to the stretching vibration of calcium alginate. The optical property of the composite was determined using UV−vis absorption spectra and the corresponding fluorescence (FL) intensity as in Figure 2c,d.

### 3.2. The Removal Efficiency of ARB Using ZnO/AC Alginate Beads

**Figure 3:**
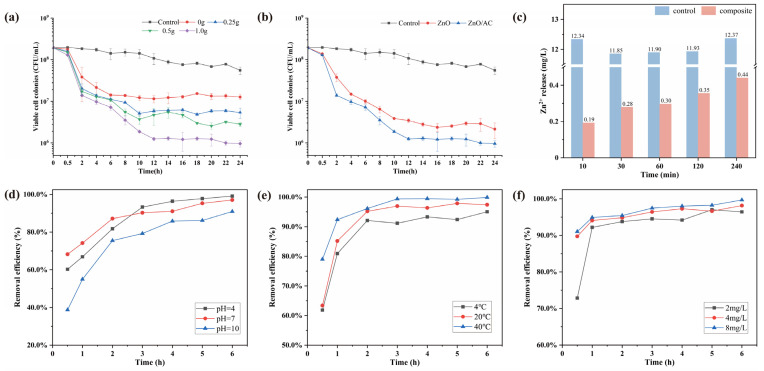
The curves of viable cells in solution after exposure to different proportions of (**a**) ZnO or (**b**) AC in beads (The proportions of ZnO is 2 g/L). (**c**) The amount of Zn^2+^ released from the composite beads over 4 h (control = 2 g/L ZnO particles; composite = relative concentration of 2 g/L ZnO in composite beads). (**d**–**f**) Effects of pH, temperature, and the concentration of antibiotics on removal efficiency.

The ARB removal efficiency using alginate beads with different ZnO contents of 0 g, 0.25 g, 0.5 g, and 1.0 g (≈0, 0.5, 1, 2 g/L) was shown in Figure 3a. In the control group, the bacterial activity decreased slightly without any treatment and was ultimately stabilized around 10^8^ CFU/mL after 24 h. The beads without ZnO only adsorbed in the early stage and gradually reached an adsorption equilibrium of 1.13-log removal within 6 h, consistent with previous studies of AC hydrogel [30,31]. As illustrated in Figure 3a, the removal efficiency increased with the increase in ZnO concentration, and the 2 g/L ZnO composite exhibited the highest removal efficiency of 2.3-log with the longest duration of 12 h. Given that the beads with ZnO adsorbed ARB and also caused the inactivation of ARB. The removal efficiencies of 1 g/L and 2 g/L of ZnO were significantly different from 2 h to 24 h (*p* < 0.05) and showed highly significant differences from 6 h to 24 h (*p* < 0.01). In addition, the initial concentration of bacteria was much higher, and 2 g/L ZnO-alginate beads were more durable, with a removal efficiency of 99.5% among those previous studies [25,26]. For the same content of ZnO, ZnO/AC alginate beads in this study also displayed excellent efficiency in removing ARB compared to the others [23]. As shown in Figure 3b, adding activated carbon increased the removal efficiency of 0.35–0.49-log.

The removal efficiency above seems to depend on the concentration of ZnO, whereas, for practical use, the maximum allowable concentration of Zn^2+^ in water is 3–5 mg/L [32]. Hence, the release of zinc ions was determined by ICP-MS, and Figure 3b showed the amount of Zn^2+^ was 0.44 mg/L over 4 h, which was far below the limit and safe for water use. The low leaching of ZnO is mainly attributed to the sustained release property of calcium alginate [33]. Overall, our results suggested that 2 g/L ZnO of alginate beads exhibited a remarkable removal efficiency and could be an ideally packed material in subsequent fixed bed experiments.

Various environmental factors may play an essential role in the removal difference in practical application. As shown in Figure 3d~f, different groups of pH, temperature, and the concentration of antibiotics were considered to determine the stability of ZnO/AC alginate beads. Based on the results, environmental conditions of acidity (pH = 4), high temperature (40 °C), and high antibiotic concentration (8 mg/L) led to higher removal efficiency of ARB. However, the total removal efficiency became similar (90.9–99.9%) when the composite reached plateaus. Thus, the ZnO/AC alginate beads could maintain high removal efficiency at different pH, temperature, and antibiotic concentrations.

### 3.3. Application of ZnO/AC Alginate Beads in Fixed Bed

#### 3.3.1. Removal Efficiency of ARB under Different Parameters

**Figure 4:**
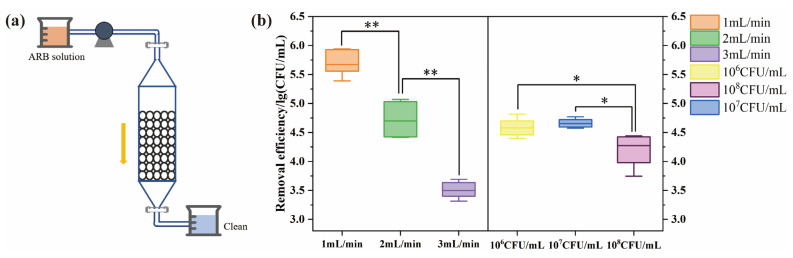
(**a**) Schematic representation of fixed bed column. (**b**) The removal efficiency of ARB with different flow rates (1, 2, 3 mL/min) (ARB samples of 50 mL were filtered with an initial bacterial concentration of 10^8^ CFU/mL) and initial concentrations (10^6^, 10^7^, 10^8^ CFU/mL), (Flow rate was 2 mL/min; * for *p* < 0.05; ** for *p* < 0.01).

The application of ARB removal was investigated using columns packed with the composite, while experiment groups were designed using operational parameters, such as flow rate and initial concentration. As shown in Figure 4b, the minimum flow rate exhibited the best efficiency with only 10^2^ CFU/mL residue in the effluent. In addition, the bacteria concentration at the outlet increased by about 1-log progressively with increased rates. Our results also showed significant differences (*p* < 0.01) among different flow rates, probably because of the shorter contact and reaction time between the cell membrane and beads surface under faster flow rates [34]. Meanwhile, fixed bed systems of different initial concentrations (10^6^, 10^7^, 10^8^ CFU/mL) were studied with the same bed depth and a flow rate of 2 mL/min. As shown in Figure 4b, all three groups showed removal efficiency of about 4.5-log, and the lower concentration resulted in fewer colonies of ARB in the effluent. In addition, bacterial concentrations of 10^6^ and 10^7^ CFU/mL were both significantly different (*p* < 0.05) from the concentration of 10^8^ CFU/mL. Zhao reported that the ceramic disk filter could remove 99.99% [35] of *E. coli*, while Huang reported that a filter coated with nano ZnO removed 99.89% of *E. coli* [36]. Our findings showed that a fixed bed with a flow rate of 1 mL/min and initial concentrations of 10^6^ and 10^7^ CFU/mL exhibited higher bacterial removal, with a removal efficiency of 4.19-log~5.69-log. The higher removal efficiency compared with the static experiments might be caused by a comprehensive effect of inactivation, adsorption, and interception in the column.

#### 3.3.2. Removal Efficiency of ARGs under Different Parameters

**Figure 5:**
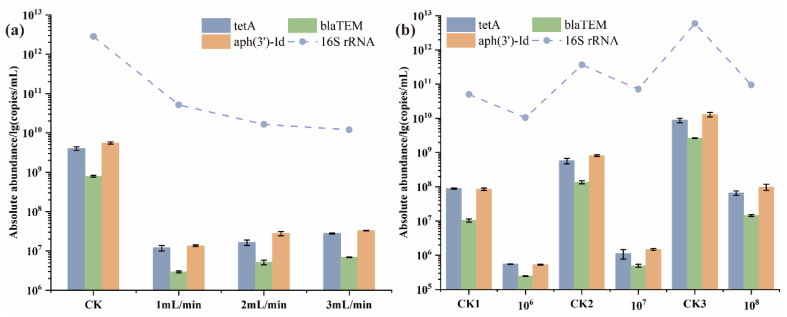
The absolute abundance of ARGs (*tet*A, *bla*_TEM_, and *aph*(3′)-Id) and 16S rRNA at the outlet with different (**a**) flow rates (1, 2, 3 mL/min) (CK = control group; ARB samples of 50 mL were filtered with an initial bacterial concentration of 10^8^ CFU/mL) and (**b**) initial concentrations (10^6^, 10^7^, 10^8^ CFU/mL) (CK1,2,3 = control groups at different concentration and flow rate was 2 mL/min).

The absolute abundance of ARGs was analyzed through qPCR. As shown in Figure 5a, three ARGs of *E. coli* HB101 were reduced to a certain extent, among which the absolute abundance of ARGs with flow rates (1, 2, 3 mL/min) decreased by 2.06–2.61-log. For different rates, our studies found that the removal efficiency of three genes decreased steadily. Regarding the inlet concentration, the ARGs removal efficiency of 10^7^ CFU/mL (2.71-log, 2.44-log, and 2.74-log showed significant differences with the concentrations of 10^6^ CFU/mL and 10^8^ CFU/mL. These differences might be caused by the proportion of bacteria captured on the composite surface based on the abundance of bacteria monitored using the 16S rRNA gene [37]. According to previous studies, the ARGs removal efficiency of chlorination and UV treatments [12,13] was often within 80%. This fixed bed seemed highly effective, with more than 95% ARGs removal efficiency under different parameters. Thus, the filter system showed high efficiency in removing both ARB and ARGs, which can be considered a favorable alternative to purifying wastewater.

#### 3.3.3. The Performance Analysis of Fixed Bed

**Figure 6:**
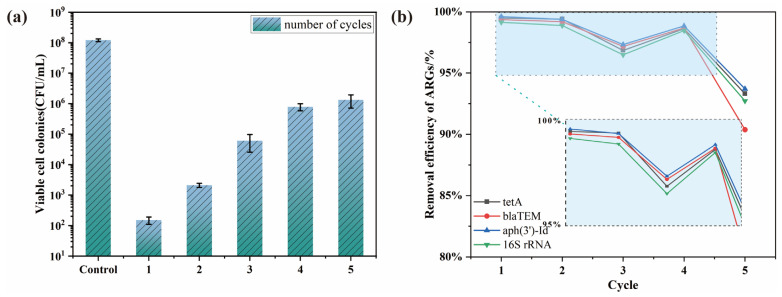
(**a**) The number of colonies of *E. coli* HB101 after filtration in five cycles; (**b**) The proportion of removal efficiency (%) of ARGs after filtration in five cycles. (ARB Samples = 50 mL, initial bacterial concentration = 10^8^ CFU/mL, and flow rate = 1 mL/min).

The performance of the fixed bed was expressed through the breakthrough curve, which reveals the time-dependent variation of bacterial concentration at the outlet [38,39]. Three kinds of bed curves (10 cm, 20 cm, and 30 cm) with a concentration of 10^6^ CFU/mL and flow rate of 1 mL/min were described in Figure S2. The breakthrough curves moved from left to right with the increase in bed depths, showing that penetration and saturation time increased, which led to more removal of ARB and ARGs. Moreover, the slope of the breakthrough curves became lower, suggesting that the saturation point reached slowly at higher bed depths. As shown in Figure S2, the breakthrough point and the saturation point were highly dependent on the bed depths. The results showed that the breakthrough point increased from 75 min to 150 min, and the saturation point increased from 400 min to 1000 min when the depths increased from 10 cm to 30 cm. The AEC of material was indicated by 4.25, 4.07, and 3.74, respectively. Compared to the previous study [40], this performance was superior to a fixed bed with zirconium-caged activated biochar alginate beads in which the saturation point was 680 min for 30 cm bed height. And beyond that, the durability of this fixed bed was examined through the removal efficiency of *E. coli* HB101 and ARGs (*tet*A, *bla*_TEM_, *aph*(3′)-Id) in five cycles (Figure 6a,b). The ARB removal efficiency reached 6-log at first, and the removal rates decreased gradually by 1-log before the fourth cycle. In the subsequent two cycles, the removal efficiency gradually stabilized to around 2-log. As depicted in Figure 6b, the efficiency decreased with the increase in the cycle and then with more than 90% removal of ARGs at the last operation cycle. Based on the durability analysis, the composite beads in fixed beds were reusable for removing ARB and ARGs.

### 3.4. Mechanism of ARB Removal

#### 3.4.1. Morphology Observation Using SEM

As indicated using SEM (Figure S3), the control group showed normal morphology of *E. coli* HB101, a rod shape with an intact and smooth membrane. In contrast, after treatment for 30 min, the membrane became wrinkled, and ZnO particles adhered to the surface. After being exposed for 120 min, obvious deformation of cell walls was observed (Figure S3c). The morphology was basically changed, as well as cell lysis and cytoplasmic leakage in high magnification. These results imply that ZnO assembled by nanosheets can cause physical damage when attached to the surface, and the release of Zn^2+^ will form *E. coli* HB101/ZnO aggregation, which accelerates cell death [41].

#### 3.4.2. Investigation of Cell Damage by CLSM

**Figure 7:**
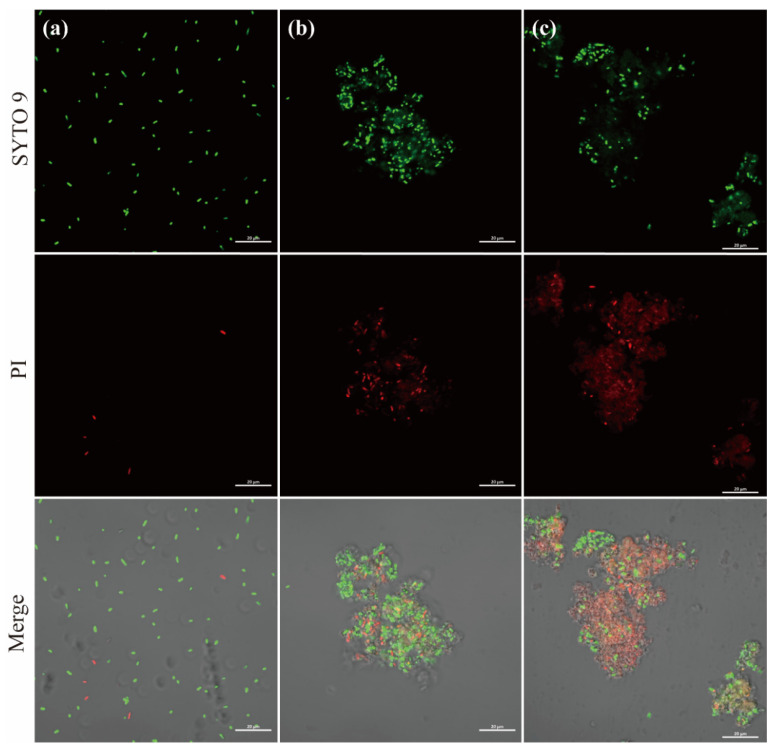
Confocal laser scanning microscopy (CLSM) images of *E. coli* HB101 after treatment for different times. (**a**) 0 min; (**b**) 30 min; (**c**) 120 min. (The green fluorescence represents viable cells; The red fluorescence represents dead cells.)

Two fluorescent nucleic acid dyes of different penetrability were performed to investigate the bactericidal mechanism. In this method, SYTO 9 stains viable cells and cytoplasm in green fluorescence, while PI stains nucleic acids of dead bacteria in red fluorescence. As shown in Figure 7a, the untreated *E. coli* HB101 showed green fluorescent rod-shaped and evenly dispersed spots with few red spots. This could be because of its natural death in the absence of nutrients. In contrast, after 30 min, bacteria appeared in agglomeration, which contributed to the depolarization of the cell membrane [42]. Moreover, the easier penetration of exogenous ROS into cells facilitated oxidative stress response and cell damage [43]. As illustrated in Figure 7b,c, the increased intensity of red fluorescence indicated a considerable number of bacterial death and serious damage to cell integrity after exposure for 120 min.

#### 3.4.3. Lipid Peroxidation and Oxidative Stress Response

**Figure 8:**
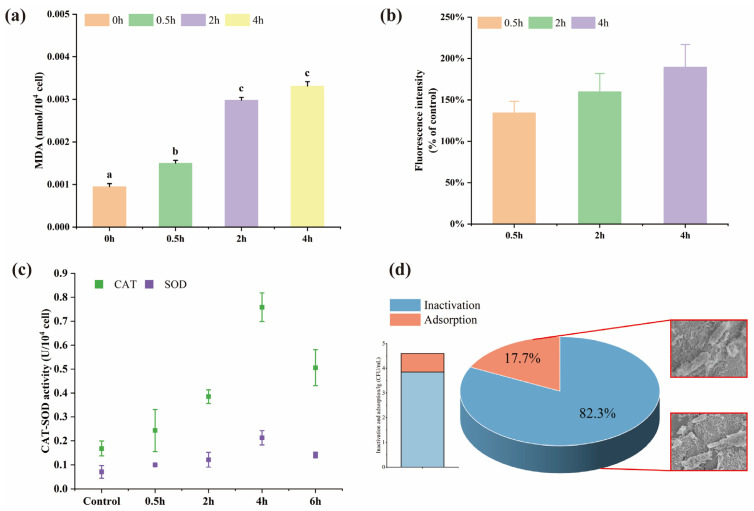
(**a**) Lipid peroxidation. Columns marked by the same letter are not significantly different (*p* < 0.05); (**b**) ROS; Different color bars represent different exposure times. (**c**) CAT-SOD activity; (**d**) Contribution of inactivation and adsorption.

Lipid peroxidation reaction is an important indicator when generated ROS attacks cell membranes [44] and can be quantified by the concentration of the major product MDA [45]. From Figure 8a, the degree of lipid peroxidation of ARB was time-dependent and increased after exposure to composite beads over a long period. In addition, a significant (*p* < 0.05) increase in the degree of lipid peroxidation of ARB was observed after treatment for 120 min. The highest concentration was observed at 4 h for 0.0033 nmol/10^4^ cells, and this correlated trend also occurred in intracellular ROS production (Figure 8b).

FCM was employed to measure the fluorescence intensity of DCFH-DA, which was positively related to the intracellular ROS content. As time passed (Figure 8b), the fluorescence intensity increased significantly compared to the control group, with no significant difference at time intervals. ROS generally maintains a dynamic equilibrium and regulates intracellular redox homeostasis in the respiratory process [46]. However, our results indicated that the intracellular ROS of ARB was out of balance and that the excessive ROS might induce cells into an oxidative stress state. Intracellular ROS was mainly produced due to cytotoxic response when ZnO particles or Zn^2+^ entered the cells [47]. Previous studies have also proven that ZnO solution produced a significant quantity of ROS even in dark conditions [48]. Moreover, the enzyme levels of SOD and CAT depicted the antioxidant activity against ROS. As shown in Figure 8c, both antioxidant enzyme activities were significantly higher than those of the control before 4 h. However, these activities decreased at 6 h, indicating that SOD and CAT were in high expression at the beginning to maintain ROS balance until the antioxidant defense system collapsed under the accumulation of ROS.

#### 3.4.4. Contribution of Inactivation and Adsorption

The inactivation capacity of the ZnO/AC alginate beads was greater than its adsorption (Figure 8d). The results showed that each AC bead could adsorb 3.95 × 10^4^ CFU/mL, while the number of colonies decreased significantly on ZnO/AC beads (7 × 10^3^ CFU/mL). It indicated that the contribution of inactivation and adsorption upon each composite bead was around 17.7–31.6% and 68.4–82.3%, respectively. To visually characterize the morphology of ARB with and without ZnO, SEM images of bacteria on two kinds of material surface were acquired. The cell membrane was intact when exposed to AC beads. In contrast, bacterial fragments were observed in ZnO/AC beads which suggested that ZnO caused the inactivation of bacteria.

## 4. Conclusions

In summary, our research provided efficient and stable ZnO/AC alginate beads with less environmental impact. A designed fixed bed system of the composite can significantly remove ARB and ARGs from highly contaminated wastewater. It was demonstrated that the system exhibited the highest removal efficiency of ARB (5.69-log) and ARGs (99.5%) when the flow rate was 1 mL/min, and the initial bacterial concentration was 10^7^ CFU/mL. In addition, after exposure to the composite beads, the direct contact or release of ZnO, lipid peroxidation, and oxidative stress caused by generated ROS would collectively lead to membrane disruption and cell death.

Considering the DBPs production during conventional disinfection, this study has put forward a novel biopolymer and its application system that can be deemed as a potential pattern in water purification, especially for point-of-use in areas with poor sanitary conditions. Moreover, antimicrobial resistance, which threatens public health, has always been ignored in wastewater treatment. Therefore, the composite beads with high effectiveness of adsorption and disinfection combined have overcome the limitations of removing ARB and ARGs.

## Data Availability

Not Applicable.

## Supplementary Materials

The following supporting information can be downloaded at: https://www.mdpi.com/article/10.3390/polym15092215/s1, Table S1: The standard curves of Qpcr; Figure S1: (a) SEM image and (b) enlarged view of the as-prepared hierarchical ZnO nanoflowers. (c) The antibacterial property of synthesized ZnO (ZnO* = ZnO nanoflowers); Figure S2: Breakthrough curves of the composite beads during ARB removal at different bed depth (Initial bacterial concentration = 106 CFU/mL; flow rate = 1 mL/min); Figure S3: SEM images of *E.coli* HB101 after treatment for different time. (a) 0 min, (b) 30 min and (c) 120 min.
