# Supplementary material for: 3D ZnO/Activated Carbon Alginate Beads for the Removal of Antibiotic-Resistant Bacteria and Antibiotic Resistance Genes
Source: Polymers (Basel). 2023 May 7;15(9):2215. doi: 10.3390/polym15092215 (PMC10180892; doi:10.3390/polym15092215)
Supplement: Supplementary file 1 [file polymers-15-02215-s001.zip › polymers-2388326-supplementary.pdf]

Table S1. The standard curves of qPCR

Gene	Standard curve	R^2	Amplification efficiency/%
tetA	y = -3.0876x + 39.05	0.9985	110
bla_{TEM}	y = -3.1115x + 38.626	0.9904	109
aph(3')-Id	y = -3.2168x + 39.788	0.9938	105
16S rRNA	y = -3.1678x + 45.778	0.9947	107

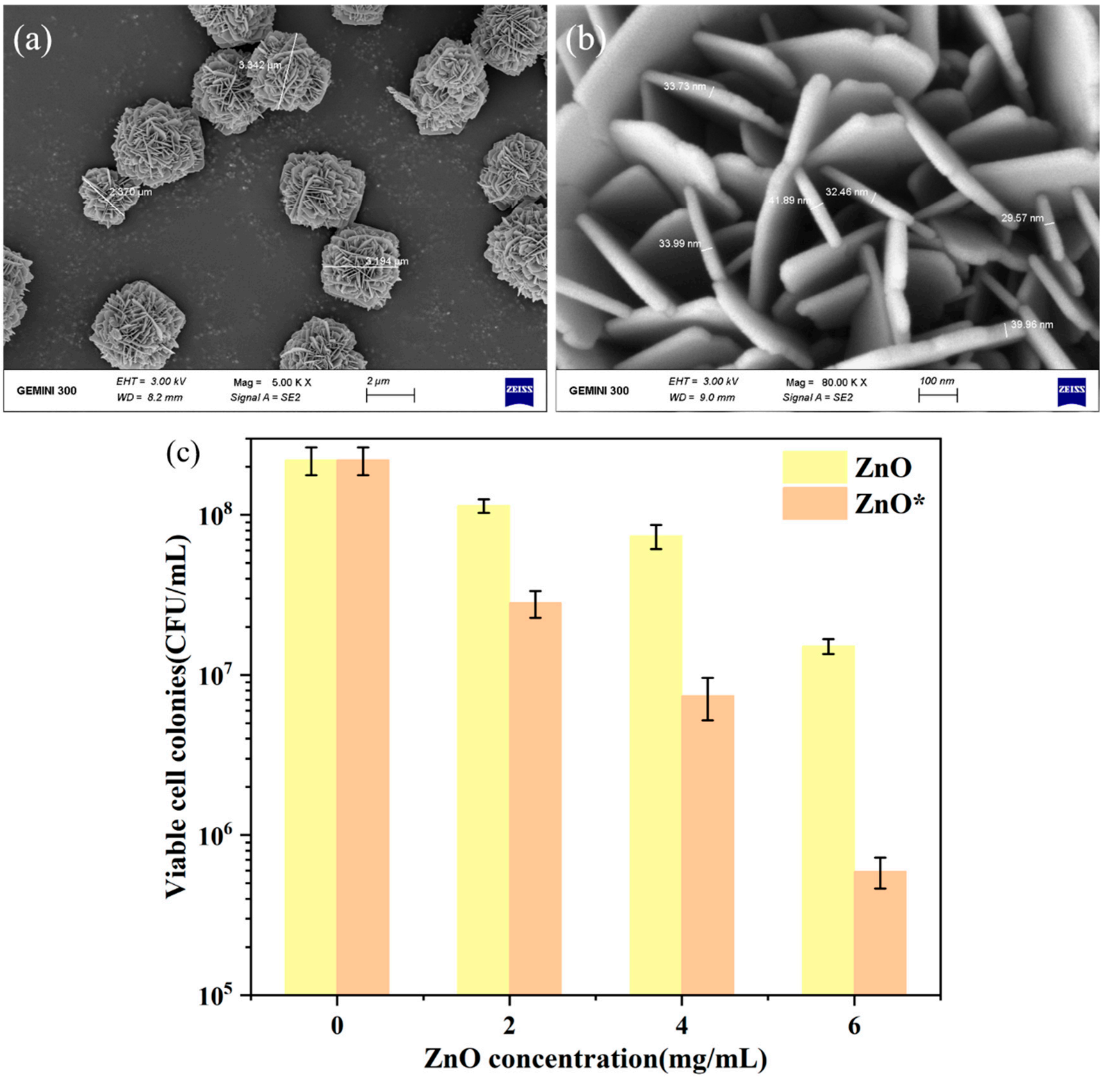

Figure S1. (a) SEM image and (b) enlarged view of the as-prepared hierarchical ZnO nanoflowers. (c) The antibacterial property of synthesized ZnO (ZnO* = ZnO nanoflowers).

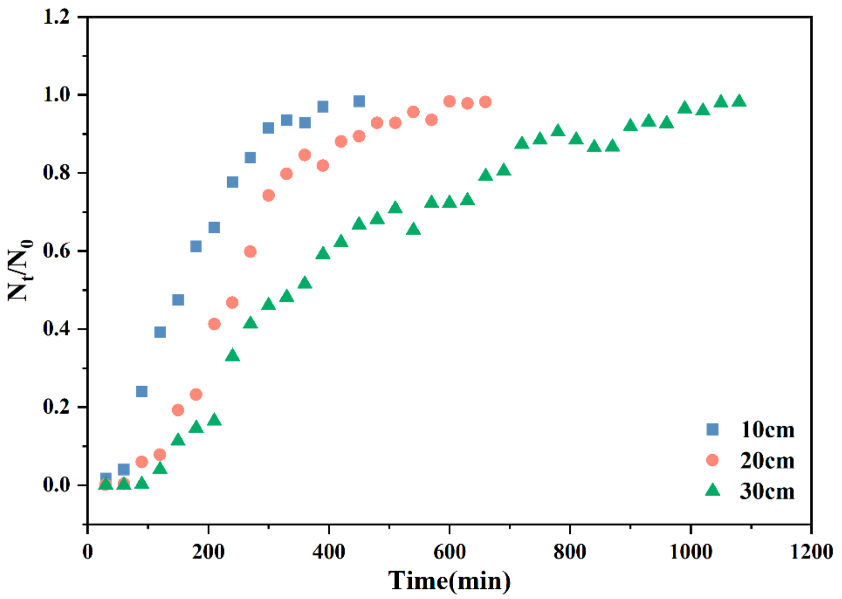

Figure S2. Breakthrough curves of the composite beads during ARB removal at different bed depth (Initial bacterial concentration = 106 CFU/mL; flow rate = 1 mL/min);

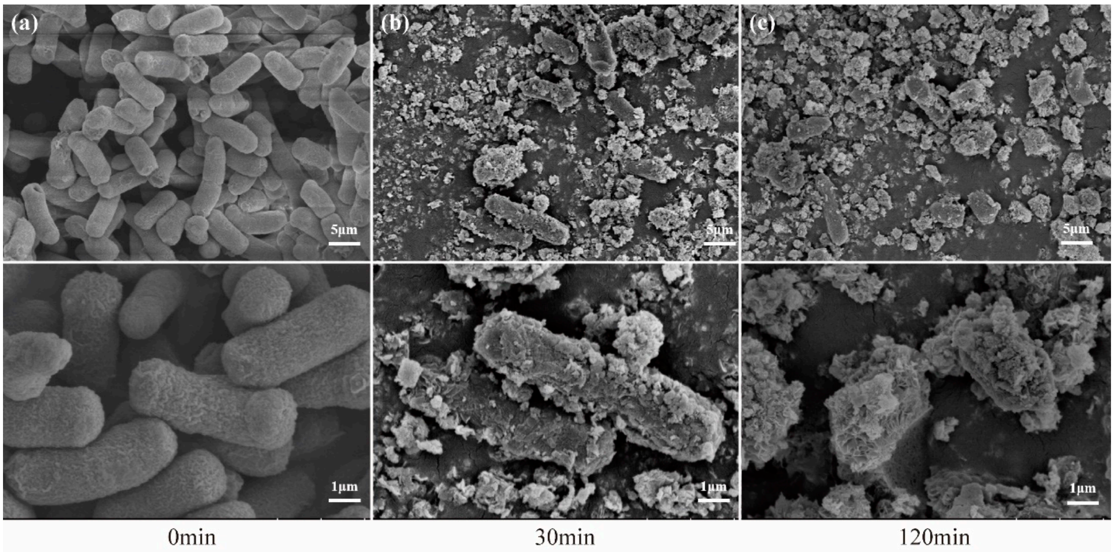

Figure S3. SEM images of *E.coli* HB101 after treatment for different time. (a) 0min, (b) 30min and (c) 120min.
